# Outcome related to impact on daily living: preliminary validation of the ORIDL instrument

**DOI:** 10.1186/1472-6963-7-139

**Published:** 2007-09-02

**Authors:** David Reilly, Stewart W Mercer, Annemieke P Bikker, Tansy Harrison

**Affiliations:** 1 AdHom Academic Departments, Centre for Integrative Care, Glasgow Homœopathic Hospital, 1053 Great Western Road, Glasgow G12 OXQ, Scotland, UK; 2 General Practice and Primary Care, Division of Community-based Sciences University of Glasgow, Glasgow G12 9LX, Scotland, UK

## Abstract

**Background:**

The challenge of finding practical, patient-rated outcome measures is a key issue in the evaluation of health care systems and interventions. The ORIDL (Outcome in Relation to Impact on Daily Living) instrument (formerly referred to as the Glasgow Homoeopathic Hospital Outcomes Scale or GHHOS) has been developed to measure patient's views of the outcome of their care by asking about change, and relating this to impact on daily life. The aim of the present paper is to describe the background and potential uses of the ORIDL, and to report on its preliminary validation in a series of three studies in secondary and primary care.

**Methods:**

In the first study, 105 patients attending the Glasgow Homoeopathic Hospital (GHH) were followed-up at 12 months and changes in health status were measured by the EuroQol (EQOL) and the ORIDL. In the second study, 187 new patients at the GHH were followed-up at 3, 12, and 33 months, using the ORIDL, the Short Form 12 (SF-12), and the Measure Yourself Medical Outcome Profile (MYMOP). In study three, 323 patients in primary care were followed for 1 month post-consultation using the ORIDL and MYMOP. In all 3 studies the Patient Enablement Instrument (PEI) was also used as an outcome measure.

**Results:**

Study 1 showed substantial improvements in main complaint and well-being over 12 months using the ORIDL, with two-thirds of patients reporting improvements in daily living. These improvements were not significantly correlated with changes in serial measures of the EQOL between baseline and 12 months, but were correlated with the EQOL transitions measure. Study 2 showed step-wise improvements in ORIDL scores between 3 and 33 months, which were only weakly associated with similar changes in SF-12 scores. However, MYMOP change scores correlated well with ORIDL scores at all time points. Study 3 showed similar high correlations between ORIDL scores and MYMOP scores. In all 3 studies, ORIDL scores were also significantly correlated with PEI-outcome scores.

**Conclusion:**

There is significant agreement between patient outcomes assessed by the ORIDL and the EQOL transition scale, the MYMOP, and the PEI-outcome instrument, suggesting that the ORIDL may be a valid and sensitive tool for measuring change in relation to impact on life.

## Background

The challenge of finding practical, patient-focussed, clinically-relevant outcome measures is a key issue in the increasing call for patient-centred care and clinical governance in health systems around the world [1,2]. However, there are relatively few patient-rated outcome measures that seek to measure change from the patients' perspective, using simple concepts such as change in main problems or symptoms and well-being [3,4]. Furthermore, it is difficult to know from these measures how meaningful any reported changes are in terms of impact on the individuals' daily life.

The ORIDL (Outcome in Relation to Impact on Daily Living) instrument measures patients' views of the outcome of care [5]. Created initially for evaluating experimental clinics [6], its increasing adoption under its former name, the Glasgow Homoepathic Hospital Outcome Scale (GHHOS), in a number of centres [7-11] and studies [12-17] has created a need to study its validity which has not previously been explored. It differs from many other scales by asking about change directly, and by relating outcome to the effect of the intervention on impact on daily life. In the present paper we report on a series of studies in which we have had the opportunity to compare the ORIDL with three validated measures in secondary (integrative care) and primary (conventional care) settings.

## Methods

The rationale behind the development of the ORIDL was to base the instrument on the type of dialogue that the doctor (or other health care professional) and patient would have to evaluate outcome in the clinical encounter. For example, a dialogue may be as follows:

Doctor: Well, was there any effect from what we did?

Patient: Yes, I think it helped...

Doctor: Yes, but was it a useful effect? Can you give me an example?

Patient: Yes, it was really useful, I was able to get about more and go to the shops.

Doctor: Really? And is that a major change, a really marked benefit, or is it less than that?

Patient: No, this is really marked, it's the best thing I've had for the problem. I'm not saying It has cured it or anything, I mean I still have the problem, but I could live with it now.

The aim was to embed the essence of these dialogues in the design of the instrument, but allow it to be standardised in a way that would allow a questionnaire based version of the scale to measure the participants' opinions on the effects of the intervention (not their view on the natural progression of the health problems being considered). The validation studies took place in the outpatient unit of GHH, an NHS-funded Centre for Integrative care, and in primary conventional care in an area of high deprivation in Glasgow. Ethical approval was obtained for all 3 studies from the West of Glasgow University NHS Trust. Written consent was obtained from all patients. In the GHH study 1, four senior doctors took part and the participants were adult out-patients attending the GHH for various periods of time (see ref 12 for details). In GHH Study 2, nine doctors took part (not including 2 of the 4 senior doctors who took part in study 1) and the participants were all new outpatients above 12 years of age whom the doctors saw in the 3 months from September to December 2002, as reported previously [14]. In study 3, five GP Principals took part working in the same Practice, and the participants were adult patients registered with the practice.

**Figure 1:**
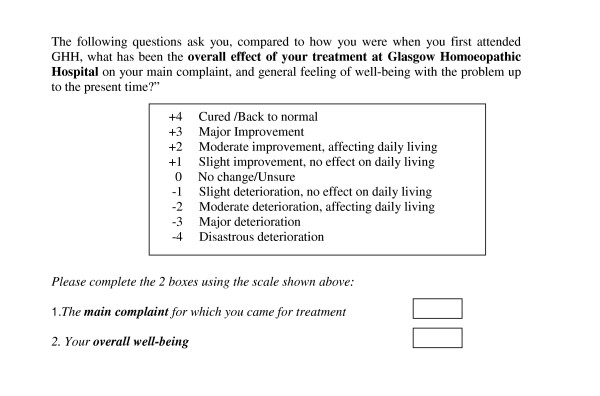
ORIDL format in study 1.

The ORIDL offers nine options for the participant to choose. In study 1, the format used to elicit patients' views was as shown in Figure 1.

**Figure 2:**
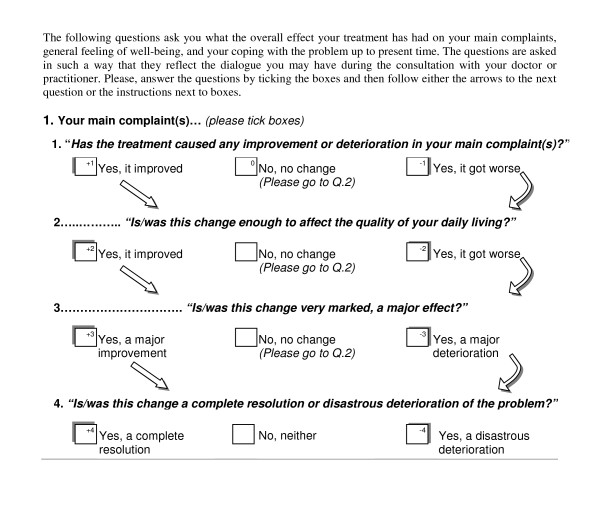
ORIDL format in study 2.

In study 2, a different format was used to elicit patients' views on these 9 options, as shown in Figure 2. This question was then repeated for 'your overall well-being'.

In study 3, the format used in study 1 was adopted (Figure 1), but modified to 'the overall effect of your treatment by your GP' as it was a primary care study of general practitioners.

A score of 2 or above was used as a 'ORIDL threshold' score as the wording at this level records an effect on quality of daily living as perceived by the patient.

### Choosing timings and targets

The target and timing of use of the ORIDL is chosen by the participants and the context, and so reporting needs to make clear what choices were used. We have used ORIDL in the current studies for patients' views. It is less explored for recording practitioners own views.

### Presenting results

*Individual *results can be recorded in the medical casenotes as need be, perhaps beside the intervention they refer to, or as overall results. This has been useful in clinical practice at the GHH as a 'shorthand' code allowing the care team to share a sense of the value or otherwise of interventions. *Group results *can be presented graphically to show the range of results for a group or cohort of patients, as reported in the present paper.

### Procedure

Immediately before their consultation, the patients were asked to complete the EQOL [18] (Study 1, GHH) or the SF-12 [18] (Study 2; GHH) and during the consultation (with the practitioner) they did the initial MYMOP [3] (Study 2; GHH, Study 3; Primary Care). After the consultation they completed demographic questions and put the forms in a sealed collection box. In Study 1, the patients were posted a follow up questionnaire containing the EuroQOL and ORIDL (main complaint and well-being) at 12 months after baseline assessment. In Study 2, the patients were posted follow up questionnaires at 3 months, 12 months and 33 months after initial consultation (baseline assessment) containing the SF-12, MYMOP, and ORIDL. In Study 3, patients were posted a follow up questionnaire at 3–4 weeks after contact consultation (baseline assessment). In studies 1 and 2, non-responders received two postal reminders, in study 3, 1 postal reminder followed by a telephone call.

### Validated measures used to compare with ORIDL

The Measure Yourself Medical Outcome Profile (MYMOP) has four items which are completed during the consultation [3]. The patients choose (with regard to their main problem) up to two main symptoms and one activity of daily living, which are scored for severity over the past week on a seven-point scale (from 0 = "As good as it could be", to 6 = "As bad as it could be"). In a similar way a general well being question is completed. By adding up the scores to all the items and dividing the total by the number of answered items an overall profile score is calculated. On the follow up questionnaire the wording of the chosen symptoms and activity are copied and scored again by the patients [3].

The EuroQOL-5D (EQOL) and the Short Form 12 (SF-12) are both widely used self-completed instruments to measure health status [18,19]. In the EQOL, five domains make up the descriptive system of the instrument; mobility, self-care, usual activities, pain/discomfort, and anxiety/depression. There are three possible responses for each domain. Results can be presented descriptively, or scores added to give an un-weighted total score or an index (weighted) score can be calculated from published tables to give a co-efficient between zero and one. In addition the EQOL has a VAS component which asks the patient to rate their current health status on a visual analogue scale from 0 (worst imaginable health state) to 100 (best imaginable health status). Finally, the EQOL also includes a transition measure, asking patients to rate their current health state against the previous 12 months (Better, Same Worse). In the SF-12, twelve questions comprise several concepts, such as physical functioning, social functioning, and bodily pain. The responses are weighted and combined to derive two summary scales: (1) the physical component score (PCS), which is an indicator of physical health (2) the mental component score (MCS), which is an indicator of mental health. The two scores range between 0 and 100 and higher scores imply better overall health.

The Patient Enablement Instrument (PEI) is a six-item measure that was developed and validated to measure the immediate outcome of consultations in primary care [20]. However, we have recently used the PEI to measure patients views on enablement over time [14] and qualitative work suggests it may be a useful outcome for patients in terms of changes in self-concept [21]. Therefore we have reported on the PEI as a longer-term outcome measure (PEI-Outcome) in the present paper.

### Participants

Study 1 (GHH) – patients completed the follow-up study at 12 months, patient details have been reported elsewhere [12,22].

Study 2 (GHH) – patients completed the follow up questionnaire at three months, at twelve months, and 33 months. Details of the patients characteristics have been previously reported [14]. An additional time points at 20 months was included using the ORIDL only.

Study 3 (Primary Care) – patients completed the follow up questionnaire at 1 month.

### Analysis

The data were analysed through the statistical package SPSS. Comparisons between the ORIDL scores with the change scores (i.e. the difference between the scores from the first and follow up questionnaires) of the EQOL, MYMOP and SF-12 was assessed by using Spearman's rank correlation coefficient. Through independent samples t-tests it was checked whether people who had scores below the ORIDL threshold (< +2) had significantly lower change scores on the MYMOP and SF-12 than the people who had scores above the ORIDL threshold (+2 and above).

## Results

### Study 1: ORIDL and EQOL scores over 12 month

105 patients consented to follow-up, and 74 returned the questionnaire at 12 months (75% response rate). Of these 74, there were 5 missing values (6.8%) for ORIDL (both main complaint and well-being). Missing values for EQOL ranged from 6.8–10.9%, (mean 9.0%).

**Figure 3:**
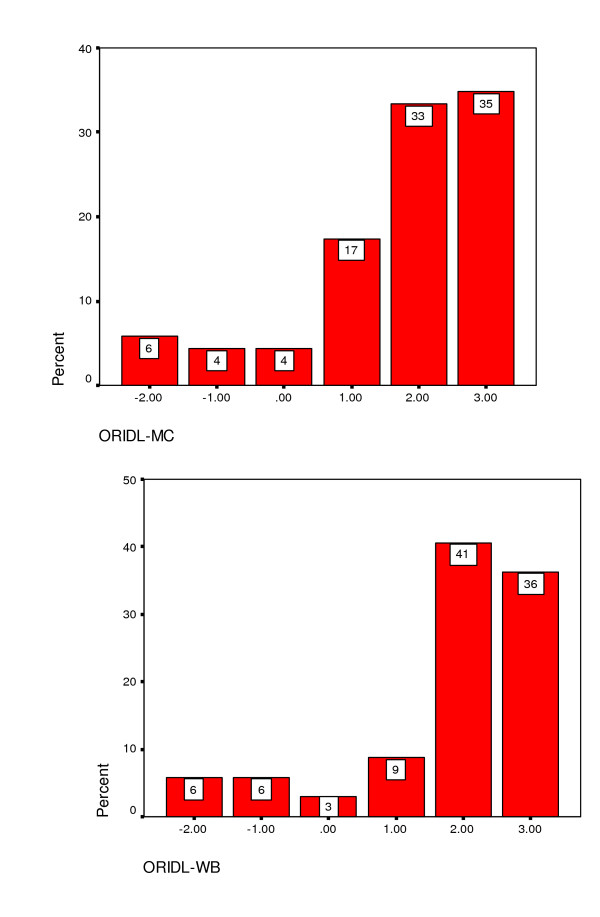
ORIDL scores over 12 months in patients attending the Glasgow Homoeopathic Hospital (Study 1).

Figure 3 shows the distribution of ORIDL scores at 12 months post contact consultation in patients attending the GHH. The majority of patients reported overall improvements in their main complaint and well-being, with some 68% and 77% reporting improvements in quality of daily living (ORIDL scores of 2+ or better) in main complaint and well-being, respectively. However, the lack of a 'ceiling effect' should also be noted, with no patient recording a score of plus 4 ('cured').

With the EQOL, small but statistically significant improvements (p < 0.001, chi-squred) between baseline and follow-up scores were observed for usual activities (13% of patients showing improvement), pain/discomfort (22% showing improvement) and anxiety/depression (13% showing improvement). No significant changes were found in mobility or self-care (results not shown). A significant but small difference in EQOL VAS score was found between baseline and follow-up (baseline; mean Health State Today 62.5, follow-up: mean Health State Today 68.6, p = 0.001) signifying a small improvement in perceived overall health status. The EQOL transition score (Health state today compared with 12 months ago) showed much larger perceived changes, with 66% of patients reporting 'better', 21 % 'same' and 13% 'worse' health state.

Table 1 shows the correlations between ORIDL and EQOL scores. No significant correlations were found between the ORIDL and the EQOL total score for the change in the 5 domains between baseline and follow-up (calculated as either a weighted or un-weighted total scores). Changes in the individual domains of the EQOL were also not significantly correlated with the ORIDL, except for a weak correlation (rho 0.25, p = 0.04) between EQOL-Mobility and ORIDL-Well-Being (results not shown). EQOL-VAS change showed a weak but significant correlation with ORIDL-wellbeing score but not with ORIDL-main complaint (Table 1). However, EQOL-transitions score (perceived change over the last 12 months) were highly correlated with both components of the ORIDL (Table 1). Similarly PEI-Outcome scores (change in enablement over 12 months) were also significantly related to ORIDL scores (Table 1). The correlation between ORIDL-main complaint and ORIDL-well being was 0.724, p < 0.001.

**Table 1:** Spearman's correlations between ORIDL and EQOL at GHH (study 1)

	**ORIDL-main complaint**	**ORIDL-well being**
Change in EQOL Score (un-weighted)	0.201 (63)	0.083 (63)
Change in EQOL Score (Weighted)	0.115 (63)	0.029 (63)
Change in EQOL – VAS	0.152 (64)	0.261 (64) *
EQOL – transition scale	0.643 *** (66)	0.546 (67)***
PEI Outcome	0.418 ***(67)	0.487 (67)***

* p < 0.05, ** p < 0.01, *** p < 0.001

### Study 2: ORIDL and MYMOP and SF-12 scores at 3 months, 12 months, and 33 months in new patients

187 new patients consented to follow-up, and questionnaire response rates were 117 (63%), 76 (41%) and 75 (40%) at 3, 12, and 33 months respectively. Missing values for ORIDL items at the different time points ranged from 0.9 – 6.4% (mean 3.4%). Missing values for MYMOP symptom 1 and well-being items ranged from 5.3–15.8% (mean 11.0%) and for SF-12 items from 4.0–22.4% (mean 13.6%).

**Figure 4:**
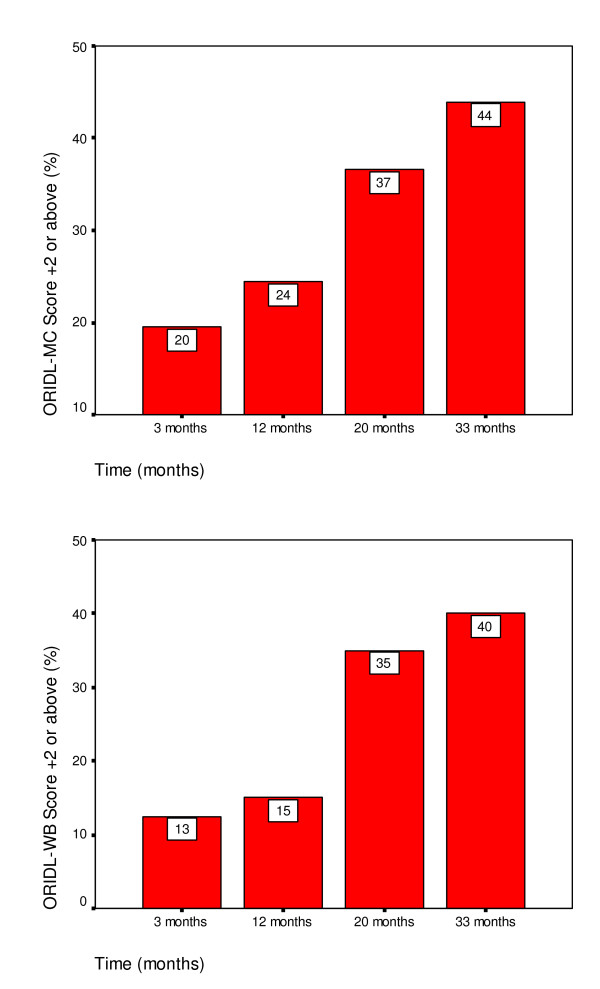
Improvements in daily living reported by the ORIDL over 33 months at GHH (Study 2).

Figure 4 shows the ORIDL scores (expressed as % scoring 2+ or above) of new patients attending the GHH followed prospectively up to 33 months. As can be seen there was a slow but steady improvement in main complaint and well-being in terms of effect on daily life over the 33 month period.

The baseline and follow-up scores at 3 months and 12 months for the SF-12 and the MYMOP have been published previously (Bikker et al 2005) and showed no changes in SF-12 scores but significant improvement in MYMOP scores. Similarly, SF-12 scores showed no significant changes between baseline and 33 months for either the physical or mental health components (results not shown). MYMOP scores did however show significant improvements (paired t-test) in scores for symptoms (baseline 4.24, 33 months 3.16, p < 0.001), well-being (baseline 3.50, 33 months 2.84, p < 0.01), and profile score (baseline 4.02, 33 months 3.18, p < 0.001).

Table 2 shows the correlations between ORIDL and MYMOP, SF-12 and PEI-outcome at 3,12, and 33 months. As can be seen, ORIDL scores were only weakly related to SF-12 change scores at all 3 time points, with few significant correlations. However, ORIDL and MYMOP change scores were much more highly correlated, showing significant relationships to each other at all time points and for all components reported (MYMOP symptom change, MYMOP well-being change, and MYMOP profile change all significantly related to both ORIDL main complaint and ORIDL well-being). There was no indication that the strength of the correlation between ORIDL and MYMOP weakened with time (i.e., correlations at 33 months were as strong, if not stronger, than at 3 months). The strongest correlations with ORIDL was generally with the change in MYMOP profile score.

**Table 2:** Spearman's correlation between ORIDL and SF-12, MYMOP, and PEI at GHH (study 2)

	**ORIDL – main complaint**			**ORIDL – well being**		
	3 months	12 months	33 months	3 months	12 months	33 months
SF-12 PCS Change	0.214* (90)	0.186 (56)	0.224 (63)	0.211* (90)	0.152 (57)	0.336** (63)
SF-12 MCS Change	0.245* (90)	0.015 (56)	0.334** (63)	0.235* (90)	0.040 (57)	0.388** (63)
MYMOP Change inProfile	0.444*** (90)	0.474*** (61)	0.499** *(68)	0.400** (99)	0.487** *(62)	0.541*** (68)
MYMOP Change in Symptoms	0.433*** (109)	0.444** *(68)	0.440 ** *(70)	0.435** *(109)	0.485*** (69)	0.446***(70)
MYMOP Change in Well-Being	0.334** (102)	0.314* (62)	0.390** (64)	0.333** (101)	0.439** *(63)	0.383** (64)
PEI Overall Outcome	0.451** (105)	0.586***(62)	-	0.528*** (104)	0.640*** (63)	-

* p < 0.05, ** p < 0.01, *** p < 0.001

Table 2 also shows that the PEI-outcome measure also correlated significantly with the ORIDL at 3 months and 12 months (PEI-outcome was not measured at 33 months).

### Study 3 (Primary Care): ORIDL and MYMOP at 1 month post-baseline

323 patients consented to follow-up, and 159 returned the questionnaire at 1 month (49% response rate). Of these 159, missing values (6.8%) for ORIDL main complaint and well-being items were 10% and 11%, respectively. Missing values for MYMOP symptom 1 and well-being items ranged from 2.0–7.0% (mean 4.7%). Missing values for PEI ranged from 2.6–5.0% (mean 4.0).

**Figure 5:**
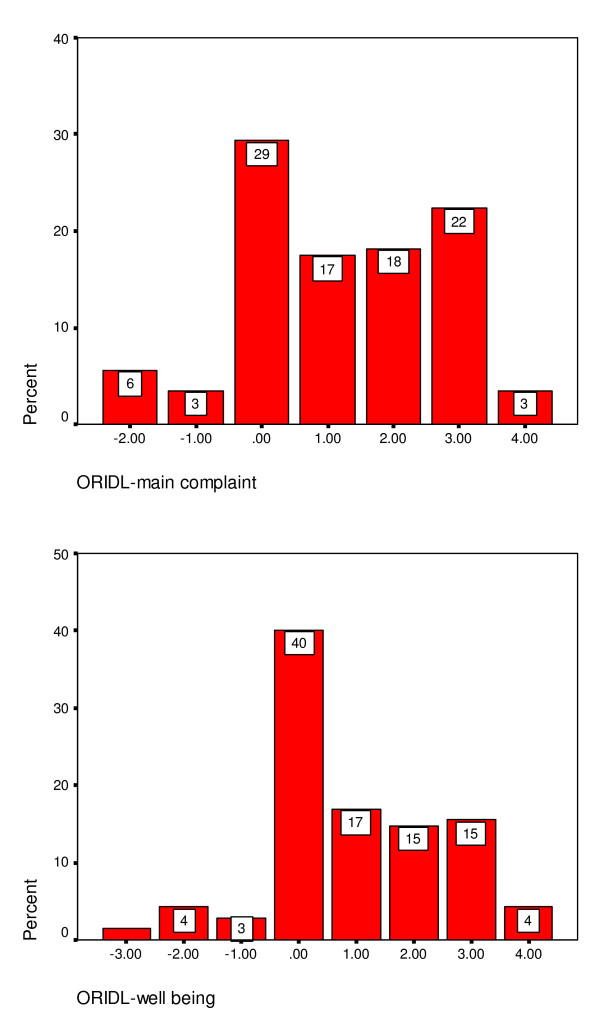
ORIDL outcomes in primary care at 1 month.

Figure 5 shows the distribution of ORIDL scores 1 month after contact consultation in primary care. The percent reporting a significant impact of daily living for main complaint is 43% and for well-being is 34%. The percentage reporting no change (a score of zero) was 29% for main complaint and 40% for well-being. This was similar to the percentage showing no change (a change score of zero) for the MYMOP (32% for symptom 1, 41% for well-being).

For MYMOP scores at baseline and 1 month significant improvements were found in symptom (baseline 4.50, 1 month 3.58, p < 0.001) and profile score (baseline 4.07,1 month 3.64, p < 0.001) but not in well-being (baseline 3.53, 1 month 3.53).

Table 3 shows the relationship between ORIDL and MYMOP and PEI in primary care. ORIDL components were highly and significantly correlated with all MYMOP change scores, with the highest correlations found between ORIDL -main complaint and MYMOP symptom change and profile change. A significant relationship between MYMOP well-being change and the ORIDL items was also found. PEI-outcome was highly correlated with the ORIDL items.

**Table 3:** Spearman's correlation of ORIDL with MYMOP and PEI in primary care (study 3)

	**ORIDL-main complaint**	**ORIDL-well being**
MYMOP Profile Score	0.510***	0.437***
MYMOP Symptom 1 Score	0.553***	0.409***
MYMOP Well-Being Score	0.358***	0.385***
PEI Outcome	0.572***	0.584***

*** p < 0.001

### The ORIDL threshold score (+2)

Scores of ≥ +2 on the ORIDL denotes a meaningful change in the outcome to the patient (i.e. sufficient to improve daily living), so it was hypothesised that patients with scores of +2 and above would have significantly higher results on the change scores of the MYMOP compare with patients with ORIDL scores below +2. As Table 4 shows, this was the case in all instances in all three studies and at all time points measured.

**Table 4:** Mean change in MYMOP profile scores in ORIDL categories 2+ or better versus less than 2+

	**ORIDL-main complaint**		P-value	**ORIDL-well being**		p-value
MYMOP Change (Profile)	**< 2+**	**2+ or better**		**< 2+**	**2+ or better**	
**GHH (3 mths)**						
Mean (SD) *n*	0.00 (1.38) *73*	1.49 (1.17) *26*	<0.001	0.14 (1.48) *73*	1.09 (1.24) *26*	<0.01
**GHH 12 mths**						
Mean (SD, n)	0.027 (1.47) *47*	1.61 (0.98) *14*	<0.01	0.38 (1.51) *53*	1.58 (0.536) *9*	<0.05
**GHH 33 mths**						
Mean (SD, n)	0.15 (1.49) *32*	1.40 (1.27) *36*	<0.001	0.32 (1.46) *40*	1.48 (1.29) *28*	<0.01
**GP 1 mth**						
Mean (SD, n)	0.25 (1.26) *106*	1.31 (1.42) *37*	<0.001	0.31 (1.36) *114*	0.99 (1.32) *28*	<0.001

## Discussion

The development of the ORIDL instrument arose from directly asking patients questions of clinical relevance: 'Did the treatment work?', 'Was it any good?', 'Is this service helping reduce suffering?' Determining outcome is always challenging, 'proof' changes as it emerges from a complex of scientific, cultural and personal factors – an 'Evidence Mosaic' – determined as much by who is asking the question, why and when, as by any abstract notion of pure science [23]. The ORIDL scale contributes by being rooted in the patient's experience of how the outcome of care has affected their daily life. As the scale (under its former name of GHHOS) has come into use in clinical and research contexts in recent years by virtue of its high face validity and practical ease of use, this paper's aim was to formalise its introduction, comment on its practical use, and take forward issues of validity.

In the present series of studies, the ORIDL instrument showed significant and moderate-high correlations with the MYMOP at all time-points examined and in both the secondary (integrative) care setting of the GHH (Study 2) and in the primary (conventional) care setting (Study 3). This is an important finding, given that the MYMOP is now a well established instrument used to chart changes in patients symptoms and well-being, and is known to be to be more responsive to change than other widely-used tools such as the SF-36 [3] and the EuroQOL [23]. Furthermore, the consistency of the relationship between the ORIDL and the MYMOP, even up to 33 months after initial baseline measurement, suggest a stability to the changes captured by the ORIDL. This is again of considerable importance, given that academic opinion is divided on the use of transition measures versus serial measures when measuring health outcome. Whereas some argue that measuring change retrospectively (as in ORIDL) is highly fallible because it depends on accurate memory of the past [24,25], others take the opposite view, with evidence to show that patients' retrospective assessments are more sensitive, and correlate better with patient satisfaction and physical and biological indicators of change in disease state [26,27]. These two opposing academic views of psychometrics and 'clinimetrics' have not been reconciled as yet, and it has been argued that both may be valid and important depending on the context [20,26]. The ORIDL scale was created by a clinician (DR) and thus reflects this direct approach of asking patients about change, as carried out by clinicians in practice. However, by anchoring the scale in the concept of improvement in daily living, it may be that the ORIDL has a meaning not found in other transition scales.

There was, as we expected, less correlation between the ORIDL and the EQOL, and the SF12, similar to the weak relationship shown between the EQOL and the MYMOP [28] and the SF-36 and the MYMOP [2], reflecting the previously reported poor sensitivity to change by these two widely used tools. The significant correlations found between the EQOL transition scale and the ORIDL, and the PEI-outcome instrument and the ORIDL are new findings, not reported previously in the literature, though the use of the PEI as an outcome measure has been reported previously [14,21].

The response rate in the present series of studies varied, with a high response rate in study one (75%), but a diminishing response rate in study two (from 60–40%) and a 49% response rate in study three. However, we have no evidence to suggest the lower response rates were related to the ORIDL. It may well relate to the length of questionnaire and the context of the study. For example, study 3 was set in a primary care centre in an area of high socio-economic deprivation, where low response rates to postal follow-up is common (S Mercer, unpublished data). The fact that the number of missing items for ORIDL was low in all three studies, and around the same or less than the other instruments (MYMOP, EQOL, SF-12), would support the face validity of the tool. However, qualitative work with patients is required to confirm this. Qualitative work is also required to allow us to establish the best way of presenting the ORIDL choices to patients. In the present work study 2 used a different format from the other two studies (as shown in the methods) but we have no information at present as to which format patients find easiest to understand.

The current data should only be seen as part of a wider picture of validation. Previous studies have found clinically coherent correlations between indicators of consultation quality and outcomes measured by ORIDL [10-12,20]. In terms of its clinical potential, any measurement scale can only give a simplified version of what goes on and cannot hope to capture and represent the richness and complexity of daily clinical care. However, as a contribution to patient-centred outcome measures, ORIDL has two key characteristics. Firstly, it is modelled on how patients and doctors already assess care in daily practice (i.e., by asking about change directly), and secondly, it anchors the outcome to the patient's assessment of significant deterioration or improvement in terms of daily living.

Further work is required to establish how many patients are needed per doctor in order to establish a reliable ORIDL score for individual doctors [25], if a comparison between doctors is desired. Further qualitative research is also required to assess patients' views on the ORIDL and this will be of major importance in trying to validate the instrument further.

### Strengths and weaknesses of the ORIDL

Overall, we feel the scale has the following strengths:

1. It is quick and simple to use and its results are easy to communicate.

2. It is generic and so flexibly adaptive to different contexts and targets.

3. It aims to link outcome to the experience of daily living.

4. It models how patients and practitioners already asses care in daily busy practice.

5. It can be used in routine clinical practice without disrupting the care process.

6. It may allow comparison across different contexts of healthcare in the degree of useful impact achieved by the intervention.

7. It establishes a useful shared language, for example, whatever the context if you say a patient experienced '+2' then others know that this was change was at least sufficient to impact on the quality of daily living.

8. It can be used without measuring a previous baseline, capturing the participants' views of any change over time.

Some of its weaknesses are:

1. It is a broad-brush measure (and so lacks detail and precision) and would often need supplemented by other measures if more detailed results are needed.

2. It is subjective, even though anchored in experience of daily living.

3. It is only as good as the participants' views, and shares all the strengths and weaknesses of ordinary clinical practice.

4. It focuses only on the participants' views of the impact of the care – other issues like 'How?', or 'Duration?, or 'Why – e.g: placebo?', or, objective verification – need to be addressed through other approaches.

5. It can be used without a measured baseline, so is subject to the bias of recall, and the shift in perception as health status changes.

## Conclusion

Given the current search for appropriate outcome measures of routine care [29,30] we think it is worthwhile to try to relate measures of outcome to the patients' view of its impact on their daily lives. Clearly further work is required to validate the accuracy of the ORIDL in assessing such change. However, our experience and data to date suggest that the ORIDL scale may prove of value in tracking routine care in clinical practice and in evaluating healthcare interventions.

## Competing interests

The author(s) declare that they have no competing interests.

## Authors' contributions

DR devised the ORIDL and co-supervised the research in studies 1 and 2. He wrote the initial version of the manuscript and contributed to modifications. He contributed in addition intellectual input at all stages. SWM carried out the data collection and analysis in Studies 1 and 3, and re-analysed part of the data in Study 2. He re-shaped the initial version of the manuscript, and led the re-writing of subsequent versions. He also contributed intellectual input at all stages. APB carried out part of the data collection in Study 2, and analysed part of the data, and contributed to writing and modifying drafts of the manuscript. She contributed intellectually, especially to the interpretation of the data. TH carried out part of the data collection in Study 2, and contributed to the final version of the manuscript. All authors read and approved the final manuscript.

## Pre-publication history

The pre-publication history for this paper can be accessed here:


